# Resting-state functional MRI of the visual system for characterization of optic neuropathy

**DOI:** 10.3389/fnhum.2022.943618

**Published:** 2022-10-18

**Authors:** Sujeevini Sujanthan, Amir Shmuel, Janine Dale Mendola

**Affiliations:** ^1^Department of Ophthalmology & Visual Sciences, McGill University, Montreal, QC, Canada; ^2^Departments of Neurology, Neurosurgery, Physiology, and Biomedical Engineering, McGill University, Montreal, QC, Canada; ^3^Montreal Neurological Institute, McGill University, Montreal, QC, Canada

**Keywords:** glaucoma, optic neuritis, traumatic optic neuropathy, resting-state fMRI, visual cortex, dorsal visual stream, ventral visual stream, LGN

## Abstract

Optic neuropathy refers to disease of the optic nerve and can result in loss of visual acuity and/or visual field defects. Combining findings from multiple fMRI modalities can offer valuable information for characterizing and managing optic neuropathies. In this article, we review a subset of resting-state functional magnetic resonance imaging (RS-fMRI) studies of optic neuropathies. We consider glaucoma, acute optic neuritis (ON), discuss traumatic optic neuropathy (TON), and explore consistency between findings from RS and visually driven fMRI studies. Consistent with visually driven studies, glaucoma studies at rest also indicated reduced activation in the visual cortex and dorsal visual stream. RS-fMRI further reported varying levels of functional connectivity in the ventral stream depending on disease severity. ON patients show alterations within the visual cortex in both fMRI techniques. Particularly, higher-than-normal RS activity is observed in the acute phase and decreases as the disease progresses. A similar pattern is observed in the visual cortex of TON-like, open globe injury (OGI), patients. Additionally, visually driven and RS-fMRI studies of ON patients show recovery of brain activity in the visual cortex. RS-fMRI suggests recovery of signals in higher-tier visual areas MT and LOC as well. Finally, RS-fMRI has not yet been applied to TON, although reviewing OGI studies suggests that it is feasible. Future RS-fMRI studies of optic neuropathies could prioritize studying the fine scale RS activity of brain areas that visually driven studies have identified. We suggest that a more systematic longitudinal comparison of optic neuropathies with advanced fMRI would provide improved diagnostic and prognostic information.

## Introduction

**FIGURE 1:**
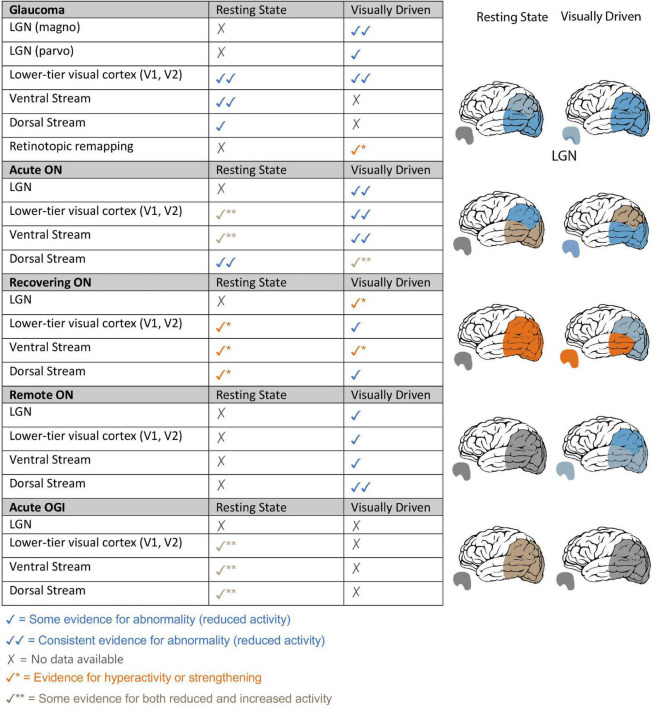
Summary of resting-state and visually driven fMRI findings in Glaucoma, Optic Neuritis and Traumatic Optic Neuropathy. **Left:** fMRI findings of glaucoma, ON and (TON-like) OGI patients are summarized based on visual areas that were investigated and themes that were revealed in our analysis of visually driven and RS-fMRI literature of each optic neuropathy. Findings from rapidly changing optic neuropathies such as ON and OGI have been split into three stages of the disease: acute – within 1 month of disease onset, recovering – 3–4 months from disease onset, and remote –1 year or more since disease onset. **Right:** pictorial representation of the fMRI findings within LGN and visual cortical areas, specifically lower visual areas, dorsal visual stream and ventral visual stream, for glaucoma patients, ON patients at different stages of the disease and OGI patients.

Optic neuropathy refers broadly to a disease of the optic nerve. It is a serious condition that may result in deficits in the visual field (partial or even full blindness). This type of injury can develop acutely or chronically. Acute optic neuropathies such as optic neuritis (ON) and traumatic optic neuropathy (TON) have a rapid onset and are typically caused by inflammation and/or trauma, whereas chronic optic neuropathies such as glaucoma are characterized by a slow onset (Behbehani, 2007). The aim of this selective review is to identify the reported optic neuropathy-related changes within the visual system as reported in resting-state functional MRI (RS-fMRI) studies, and assess the consistency between RS-fMRI and visually driven fMRI findings for each optic neuropathy. We strive to provide insights into the common and differential effects of chronic versus acute onset of optic neuropathies over the course of the disease along with central versus peripheral visual field defects on visual brain areas. A summary of visually driven fMRI results is provided for reference (Figure 1). This review is offered as a companion to the longer review of visually driven studies published in this same issue, with the same inclusion criteria (Sujanthan et al., 2022). We note that graph-theory analysis RS-fMRI papers were excluded as they have not been utilized to study all optic neuropathies we consider in this manuscript. In addition, they mainly focus on brain alternations outside the visual system, which is beyond the scope of this review.

### Resting-state functional magnetic resonance imaging

Resting-state functional magnetic resonance imaging is emerging as a powerful tool to non-invasively quantify brain activity at rest (i.e., spontaneous brain activity) of both intact visual brain regions and visual regions that have lost visual input (e.g., Cole et al., 2010). RS-fMRI allows for the study of local organization and functional connectivity (FC) of brain structures via temporal synchronization of neuronal activity. The resting BOLD (blood oxygen level-dependent) signal can be analyzed using various techniques. Below, we describe RS-fMRI analysis techniques employed in optic neuropathy studies.

Amplitude of low-frequency fluctuation (ALFF) and regional homogeneity (ReHo) are functional *segregation* methods for identifying neural networks across the whole brain, where brain regions are divided according to their presumed shared function, and provides information about local neural activity rather than FC between different regions (Lv et al., 2018). ALFF evaluates the combined BOLD signal in a region of interest by measuring the brain signal variability of each voxel in a particular frequency domain. ReHo is a method to evaluate the synchrony or “similarity” of the time course of each voxel in a region of interest to its neighboring voxels in the time domain (Lv et al., 2018). Alternatively, the following methods measure temporal synchronization between separated brain regions to assess functional *integration*. Strong FC may arise from direct or indirect anatomic connections or by way of having common input. Seed-based methods determine the brain regions closely associated with the BOLD time-series of a seed region, i.e., any brain region determined in an *a priori* manner. In comparison, independent component analysis (ICA) is more data-driven and ideally detects all functionally connected networks within the brain. However, the number of networks that can be detected is limited by the number of independent components specified. A few commonly identified RS-networks include the default mode network, medial executive network, working memory, and visual network (Lv et al., 2018). Finally, voxel-mirrored homotopic connectivity (VMHC) is a specific voxel-wise analysis method in which the synchrony between geometrically corresponding interhemispheric regions at rest is quantified. It is yet to be determined what combination of RS-fMRI analysis methods could be most sensitive to detecting optic neuropathy-related brain deficits.

## Resting-state functional magnetic resonance imaging in glaucoma

Glaucoma is a chronic optic neuropathy resulting from the loss of retinal ganglion cells. It is characterized by reduced retinal thickness, peripheral vision loss in the early stages of the disease, followed by central visual field loss, and eventual low vision or blindness (Weinreb and Tee Khaw, 2004; Jonas et al., 2017). Here, we consider the most common form, primary open-angle glaucoma (POAG). The effects of the visual deficits on the FC of cortical networks are yet to be fully understood. We selectively identified 12 RS-fMRI peer-reviewed articles with patient groups that meet our inclusion criteria and findings relevant to the themes revealed in our analysis of the *visually driven* glaucoma literature (Sujanthan et al., 2022). We aim to explore here if RS-fMRI findings reveal results similar to those obtained with visually driven protocols. We thus expected to find: (1) reduced activity in lower visual areas, especially eccentricity-specific deficits, (2) disease severity-specific alterations in brain activity; for example biased loss of activity in LGN magnocellular layers in early POAG patients, and compromised cortical representation of central visual field only in advanced patients, and (3) remapping in visual cortices with enlarged parafoveal regions and/or higher-than-normal activity in visual association and higher-level cortical areas.

As expected, RS-studies utilizing functional *segregation* methods do in fact indicate poor local specialization in regions of the visual network of POAG patients. For instance, ALFF values were lower in the cuneus (Li et al., 2014) and ReHo revealed lower synchrony between neighboring voxels in the bilateral calcarine cortex (Song et al., 2014). Likewise, functional *integration* methods noted dysconnectivity *between* lower-tier and higher-tier visual areas (Frezzotti et al., 2016; Giorgio et al., 2018). ICA and voxel-wise analyses revealed decreased FC between the primary and secondary visual cortices (Wang J. et al., 2017), and reduced interhemispheric synchronization of homotopic primary and secondary visual areas (Zhou et al., 2016; Wang et al., 2018). However, we note that there are no RS-fMRI studies that investigate retinotopy-specific functional alterations *within* primary visual areas in glaucoma patients at a fine scale. This has already been achieved in healthy controls (e.g., Dawson et al., 2013, 2016) as well as in amblyopia (Mendola et al., 2018), and could potentially identify the progression of glaucoma based on the representation of the central and peripheral eccentricities.

With regard to the higher-level visual cortex, we noted common reports of abnormally low resting connectivity in the ventral visual stream based on: (1) ALFF (Li et al., 2014; Liu and Tian, 2014), (2) ICA-based FC (Frezzotti et al., 2016), (3) cerebral blood flow-FC strength (Wang et al., 2021), (4) VMHC (Wang et al., 2018), and (5) voxel-wise analysis (Dai et al., 2013). In comparison, there are fewer reports of deficits in the dorsal stream (i.e., ReHo) (Song et al., 2014), or functional dysconnectivity between primary visual cortex and dorsal visual stream (i.e., VMHC and voxel-wise analysis) (Dai et al., 2013). While interesting, this moderate tendency for greater involvement of the ventral visual cortex was not evident in our review of studies using visually driven fMRI with glaucoma patients. Instead, that review hinted at the loss of magnocellular input – at least in early POAG – which is expected to most severely affect the dorsal visual stream. It thus remains to be understood if these tentative trends are reproduced, and exactly how such biases depend on severity.

As per our second hypothesis, the RS-fMRI literature shows that the pattern and extent of alterations are dependent on glaucoma severity. From the limited number of studies that report results in specific POAG subgroups, it can be noted that the areas in the ventral visual network show low resting FC in early POAG patients (inferior temporal gyrus and LOC) (Frezzotti et al., 2016; Wang et al., 2021), and a combination of low and high FC patterns in advanced POAG patients (Frezzotti et al., 2014). For example, FC of some areas in advanced POAG is lower (lingual gyrus), whereas others are unexpectedly higher (LOC and temporo-occipital fusiform cortex) compared to controls (Frezzotti et al., 2014). Although the abnormalities in the ventral visual stream could be consistent with the loss of central representation observed in advanced glaucoma patients, it is unclear why this effect is evident even in early POAG patients. Lastly, in comparison with visually driven fMRI, RS-fMRI studies emphasize functional alterations beyond visual areas in cortical areas proximal to the visual network from early stages of the disease (e.g., the memory and DMN networks) (e.g., Frezzotti et al., 2016).

Concerning our third hypothesis, regarding compensatory activity, RS-fMRI of glaucoma patients also reveals abnormally high brain activity, but generally in areas more distal from the visual network. For example, high resting FC can be observed within the medial executive network or other distant higher-level brain regions, as well as between these networks and the visual network (Dai et al., 2013; Frezzotti et al., 2014; Li et al., 2014; Liu and Tian, 2014; Song et al., 2014; Zhou et al., 2016; Giorgio et al., 2018; Zhang et al., 2019). This common type of observation is usually considered to reflect some unspecified state of “compensation.” Such concepts are poorly understood, but do at least document that bidirectional changes in activity are likely the result of localized damage to a complex network.

## Resting-state functional magnetic resonance imaging in optic neuritis

Optic neuritis is an acute optic neuropathy resulting from inflammation, and subsequent lesions of the optic nerve, followed by spontaneous recovery. In the acute stages of the disease (e.g., within 1 month of onset), the affected eye displays greatly delayed and decreased visually evoked potential (VEP) responses (i.e., demyelinated optic nerve), as well as poor visual acuity and color vision (Toosy et al., 2014). Subsequently, visual acuity begins to improve during the recovery phase at around 3–4 months and reaches normal or near-normal vision at 1-year from disease onset, although a reduction in retinal thickness and other abnormalities continue to exist (Beck et al., 1992). Here, we review 5 peer-reviewed articles studying RS-fMRI brain activity in ON patients that meet our inclusion criteria to explore commonalities in findings with visually driven fMRI studies (Table 1). Specifically, we would expect: (1) functional deficit within low-level visual cortex in the early stages of ON, (2) functional alterations within higher-tier areas, specifically decreased activity within the ventral visual stream (i.e., visual cortical region LOC) and a trend toward hyperactivity in the dorsal stream of early-stage ON patients, and (3) recovery of activity in LGN, visual cortex, and ventral visual stream, but persistent deficit likely in the dorsal stream (i.e., cortical area MT).

**TABLE 1:** Patient demographic and disease information from ON and (TON-like) OGI studies reviewed in this paper. Studies are listed in increasing order of average onset duration in patient population at the time of data collection. Unless stated, all studies included patients without other apparent cause of vision loss and retro geniculate diseases. ON = optic neuritis; OGI = open globe injury.

Author, Year	Number of patients	Age (Range/Mean) (± SD)	Stage & type of disease; # of occurrences	Mean time from onset at data collection
**ON**				
Shao et al., 2015	12	46.08 (± 7.91)	ON	5.3 days (± 3.2)
Huang et al., 2015	12	44.83 (± 10.71)	Acute ON	5.4 days (± 2.9)
Yan et al., 2022	21	44.83 (± 10.71)	ON	5.4 days (± 2.9)
Wu et al., 2015	15	24–56 (median: 35)	Acute; clinically isolated ON	50 days (± 25)
Backner et al., 2018	18	18 – 65	Clinically isolated ON	1–28 months (median: 3–4 months)
**OGI**				
Tan et al., 2016	18	44.6 (± 14.08)	Acute OGI	1.0 day (± 1.23)
Wang H. et al., 2017	18	44.17 (± 13.94)	Acute, *unilateral* OGI	1.0 day (± 1.23)
Huang et al., 2016	18	44.6 (± 14.08)	Acute OGI	1.1 day (± 0.4)
Ye et al., 2018	18	43.05 (± 8.79)	Acute OGI	6.6 days (± 3.4)

To our surprise, and contrary to visually driven fMRI reports, an increase in resting activity was observed in the occipital lobe immediately (i.e., within a week) after ON onset (Huang et al., 2015; Figure 1). Similarly, while visually driven fMRI studies reported reduced activity in the ventral visual stream, the majority of current RS-fMRI studies indicate increased ALFF values in many temporal lobe brain structures (Huang et al., 2015; Yan et al., 2022). However, in another study, ReHo analysis suggests an overall brain *deficit* at rest (Shao et al., 2015). Moreover, studies predominantly suggest a resting deficit within the dorsal cortical areas (i.e., fronto-parietal structures), at least during the very early stages of the disease (Huang et al., 2015; Shao et al., 2015; Yan et al., 2022). Interestingly, a different pattern is evident for the visual cortex of patients scanned around 50 days from ON onset. Now in agreement with findings reported with visually driven fMRI, lower resting connectivity is observed from the visual cortex (Wu et al., 2015). RS-FC between the visual cortex and the fronto-parietal cortex remains impaired as reported in the acute stage of ON (Wu et al., 2015). However, the relationship between the activity of extra-striate regions and patients’ visual function outcomes is unclear.

Consistent with the recovery of BOLD signals observed in the visually driven fMRI literature at 3–4 months from disease onset, one RS study with a majority of ON patients in the recovery phase revealed higher-than-normal resting FC within the visual network. For instance, abnormally high resting activity was reported in the lower (i.e., calcarine sulcus) and higher visual areas (i.e., LOC and MT) of the visual network (Backner et al., 2018). This might suggest that recovery of function is partially supported by adaptive changes in connectivity. Importantly, as expected, MT connectivity was seen to increase as the inter-eye VEP latency difference in ON patients decreased. As also concluded in the visually driven review, this suggests that the increased activity in the visual pathway is at least partly due to the reduced inflammation of the optic nerve. However, the abnormally high resting signal observed in the visual pathway raises the possibility for additional involvement by adaptive plasticity mechanisms.

Finally, the activity of LGN in ON patients has not been investigated with RS-fMRI yet, despite its ability to produce distinct levels of activity during the acute and recovery phase of ON and its potential use in objectively staging patients, as shown in visually driven fMRI. Likewise, RS-fMRI is yet to be utilized to study activity in subcortical and cortical visual regions of purely recovered ON patients. This is a particularly important group to study as it would allow us to evaluate the extent of recovery possible in areas along the visual pathway, and determine the correlation to earlier time points of visual function and BOLD measures, potentially informing the prediction of prognosis.

## Resting-state functional magnetic resonance imaging in traumatic optic neuropathy

Traumatic optic neuropathy causes acute partial or complete, unilateral or bilateral visual loss following injury to the optic nerve and demyelination of afferent visual pathways due to trauma. It can be further categorized into direct and indirect trauma, depending on how the optic nerve was affected (Miliaras et al., 2013). Like ON, substantial recovery is generally observed to take place (Singman et al., 2016). Diagnosis of TON with current neuro-ophthalmological tools may be challenging as trauma patients could have cognitive impairments. TON is typically assessed using the afferent pupillary reflex, but this is insensitive to bilateral TON (Broadway, 2012). Thus, utilizing RS-fMRI, a non-demanding imaging technique, might provide real diagnostic and prognostic value in clinical settings. Similar to our review of TON using visually driven fMRI, we found no peer-reviewed articles that studied TON using RS-fMRI. Therefore, we reviewed four papers on patients with a similar trauma, called open globe injury (OGI) (Table 1). OGI results in injury to the eye-wall and is more readily diagnosed.

Resting changes of the whole brain in OGI patients were studied using voxel-wise degree centrality (Wang H. et al., 2017), ReHo (Huang et al., 2016), ALFF (Tan et al., 2016), and VMHC (Ye et al., 2018). Indeed, functional deficit was evident within and between the two hemispheres of the brain in patients with acute lesions to the anterior visual pathway. However, the direction of the effect is quite variable and highly dependent on time since onset. For example, resting cortical activity of OGI patients within 1–2 days of onset is higher-than-normal compared to controls, especially in the primary visual cortex and precuneus as shown by degree centrality (Wang H. et al., 2017) and ALFF (Tan et al., 2016) values, as well as in lingual gyrus as shown by ALFF values (Huang et al., 2016). Interestingly, a different pattern is revealed in patients that were tested 3 days after OGI onset. Those patients display *lower* VMHC values compared to controls in the brain regions such as bilateral calcarine, lingual and cuneus and structures in the dorsal visual pathway such as the middle occipital gyrus displayed (Ye et al., 2018). This is consistent with findings from a single TON-related case report discussed in our visually driven review (Sujanthan et al., 2022). Importantly, receiver operating characteristic (ROC) curves have been used to identify brain regions based on their resting FC measures that may be used as biomarkers to distinguish OGI patients from healthy controls. With a range of analysis techniques (VMHC, ALFF and mean ReHo), ROC values regularly show areas under the curve in the range of 0.7–0.9 for the visual cortex and non-visual cortex (Huang et al., 2016; Tan et al., 2016; Ye et al., 2018), suggesting activity within the reported regions may be useful as diagnostic markers.

Taken together, OGI and TON are rapidly changing conditions that require immediate attention and treatment. It appears that initially hyperactive brain regions show deficits as the disease progresses. However, this is difficult to assert, based on the limited number of studies available, all acute cross-sectional studies. It is evident that RS-fMRI could provide valuable insight into the pathogenesis and progression of acute optic neuropathies such as TON. Therefore, if fMRI techniques are employed more frequently and ambitiously, advancement in the diagnosis or prognosis of TON might be possible.

## Discussion

Overall, when considering the visual system specifically, both RS-fMRI and visually driven fMRI studies are often consistent in the reported findings in each optic neuropathy. In chronic diseases such as glaucoma, both fMRI modalities report reduced activity in visual networks. However, RS-fMRI more commonly suggests deficits in the ventral than the dorsal visual stream, and the ventral FC pattern becomes more diverse with increasing glaucoma severity. For acute diseases such as ON and (TON like) OGI, RS-activity reveals that higher-than-normal activity is briefly observed at onset, before it ultimately shows a deficit as seen in visually driven fMRI studies. Both modalities report deficits within the dorsal pathway in ON from onset. So far, both visual streams are shown to be affected in acute OGI at rest, although no visually driven fMRI results exist.

We highlight here several important concluding points. Firstly, the resting activity of the LGN has not yet been investigated with RS-fMRI in any of the diseases we considered. This would be of particular interest given the LGN’s proximity to the optic nerve and the fact that it conveniently maintains the segregation of inputs from each eye. In addition, if resolution allows, patterns of connectivity for magnocellular or parvocellular layers would provide additional information about the pathophysiology of these diseases. In fact, this has already been reported for one fMRI study of glaucoma that used parvo- or magno-biased visual stimuli (Zhang et al., 2016). In addition, visually driven fMRI studies of ON patients have noted distinct patterns of activity within LGN during the acute and recovery stage (Korsholm et al., 2007).

Secondly, we suggest that better use of regions of interest within the visual cortex based on *a priori* hypotheses would improve the sensitivity of RS-fMRI studies to the direct effects of deafferentation. Many distinct visual areas can now be defined based on retinotopic organization or other functional characteristics. The lateral occipital region (LOC) is quite important for object perception and contains a prominent representation of the central visual field (separate from the occipital pole). Another area that may be useful to better exploit is MT, a motion-sensitive region where the magnocellular LGN inputs are expected to have a strong impact. Future studies should examine RS-fMRI activation patterns within and between multiple visual areas, as this has the potential to increase sensitivity. Probabilistic atlases now allow the location of many extrastriate areas to be estimated, even without direct mapping in each subject (e.g., Wang et al., 2015; Benson and Winawer, 2018; Rosenke et al., 2021). In fact, it is even possible to use regions of interest *within* a single retinotopic visual area to study fine-scale resting networks, and document changes in central versus peripheral eccentricities (Dawson et al., 2016; Mendola et al., 2018).

Third, we found no obvious pattern regarding the sensitivity of the different RS-fMRI analysis methods reported here, but we remain convinced that different methods are likely to reveal distinct aspects of pathology. As such, future studies that directly compare multiple analysis methods would be of enormous value. Lastly, the cross-sectional design common to most of these studies is a clear limitation. Although there is great value in following the same group of patients longitudinally, as shown by a limited number of visually driven fMRI studies (Sujanthan et al., 2022), this is rarely done in RS-studies of optic neuropathies.

In summary, we suggest that RS-fMRI data could prove increasingly valuable for characterizing optic neuropathies, particularly in the case of TON, and for managing disease progression. Given that RS-fMRI does not utilize visual stimulation or a task-induced response, it is well-suited to study vision in cognitively challenged patients or patients that are unable to maintain fixation. Moreover, the high sensitivity of fMRI data to central vision loss due to the high cortical magnification of the central field could be hugely beneficial in studying TON. In combination with other MRI modalities, including structural MRI, it could be even more powerful. For future efforts to uncover basic mechanisms of functional loss and recovery, studies that compare visually driven and RS-fMRI findings from local visual areas and closely related brain networks in the same cohort of subjects would be most useful.

## Author contributions

JM and AS conceived the idea. JM, AS, and SS developed the idea. SS wrote the manuscript in consultation with JM. All authors provided feedback and helped shape the manuscript.

## Abbreviations

ALFF: amplitude of low-frequency fluctuation
BOLD: blood oxygenation level-dependent signal
FC: functional connectivity
ICA: independent component analysis
LGN: lateral geniculate nucleus
LOC: lateral occipital cortex
MT/V5: middle temporal visual area
OGI: open globe injury
ON: optic neuritis
POAG: primary open-angle glaucoma
ReHo: regional homogeneity
ROC: receiver operating characteristic
RS-fMRI: resting-state functional magnetic resonance imaging
TON: traumatic optic neuropathy
VEP: visually evoked potentials
VMHC: voxel-mirrored homotopic connectivity.
